# Golimumab-induced lichen planus pigmentosus in a patient with ulcerative colitis^☆^

**DOI:** 10.1016/j.abd.2024.10.002

**Published:** 2025-03-13

**Authors:** Daniela Alfaro-Sepúlveda, Claudio Escanilla, Fernando Valenzuela, Dominga Peirano

**Affiliations:** Department of Dermatology, Faculty of Medicine, Universidad de los Andes, Santiago, Chile

Dear Editor,

Tumor necrosis factor alpha (TNF-alpha) inhibitors have proven efficacy in managing various immune-mediated inflammatory conditions. Five TNF-alpha inhibitors are currently available: infliximab, adalimumab, etanercept, certolizumab pegol, and golimumab. Generally, TNF-alpha inhibitors demonstrate favorable tolerability profiles but are linked to specific adverse reactions, notably cutaneous manifestations.^1^, ^2^ These include injection/infusion-related responses, skin infections, neoplasms, and immune-mediated manifestations.^2^ In this report, we present a rare case of lichen planus pigmentosus potentially triggered by golimumab administration.

A 43-year-old male patient, undergoing golimumab treatment (100 mg monthly) for a year due to a history of ulcerative colitis, presented with a progressive facial hyperpigmentation evolving over six months. The patient, with Fitzpatrick skin phototype IV, worked a desk job and had minimal sun exposure. He denied any prior history of facial or mucosal hyperpigmentation and confirmed no prior usage of topical treatments or daily cosmetic products. On physical examination, diffuse pruritic, brown-colored hyperpigmentation was observed across the face, involving the eyelids but sparing the nasal tip, devoid of scaling or erosions (Fig. 1). The patient exhibited intact mucous membranes and appendages. The dermoscopic evaluation revealed a diffuse grayish-brown hyperpigmentation with a lichenoid pattern encircling follicular openings and isolated bright white rosette-like structures (Fig. 2).

**Fig. 1:**
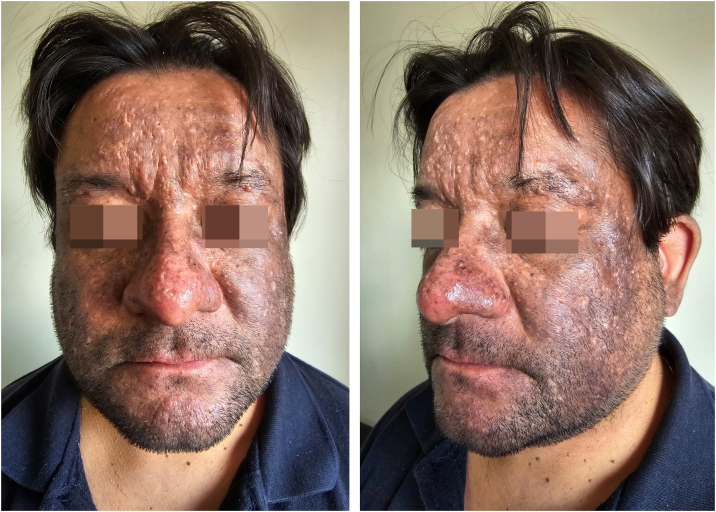
Brown-gray patches with purplish hue on the face. Note eyelids involvement.

**Fig. 2:**
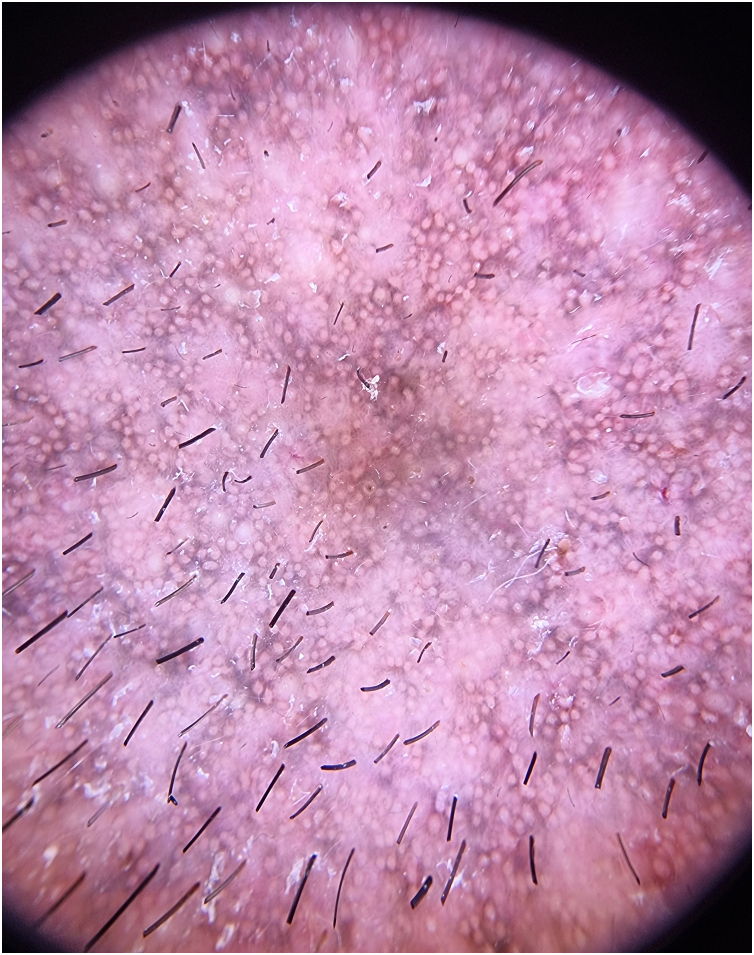
Dermoscopy. Grayish-brown hyperpigmentation with a lichenoid pattern encircling follicular openings and isolated bright white rosette-like structures.

Considering the clinical context and the progressive extension of lesions, a histopathological study was conducted, indicating findings of superficial and periadnexal lymphoplasmacytic dermatitis with a focal lichenoid interface and dermal melanosis, consistent with pigmented lichenoid dermatitis (Fig. 3).

**Fig. 3:**
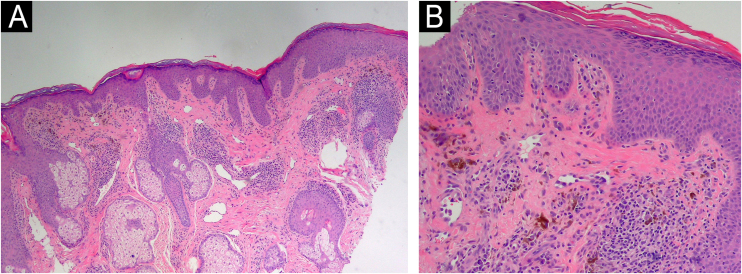
Histopathological examination of the skin biopsy shows features consistent with pigmented lichenoid dermatitis. (A) The epidermis exhibits irregular hyperkeratosis and acanthosis, with a perivascular and perifollicular lymphoplasmacytic infiltrate in the dermis, accompanied by abundant melanophages (Hematoxylin & eosin, ×100). (B) Civatte bodies are also present (Hematoxylin & eosin, ×200).

Further investigation revealed no other known triggers for lichen planus pigmentosus, such as hepatitis B or C virus, mustard oil, henna, nickel, or hair dye. A standard European patch test was performed, which returned negative results. Topical immunomodulatory therapy was initiated and suspecting a cutaneous adverse reaction secondary to golimumab usage, a reassessment by Gastroenterology was warranted. Golimumab was suspended and changed to adalimumab; this decision was influenced by the fact that, in the public health system to which the patient belongs, only TNF-alpha inhibitors (golimumab, adalimumab, infliximab) are economically covered for the treatment of refractory or severe ulcerative colitis. Currently, the patient has been on adalimumab for 4 months. The lesions have shown a slight reduction (Fig. 4); however, the pruritus has significantly diminished, despite the patient discontinuing the topical immunomodulatory therapy without medical guidance.

**Fig. 4:**
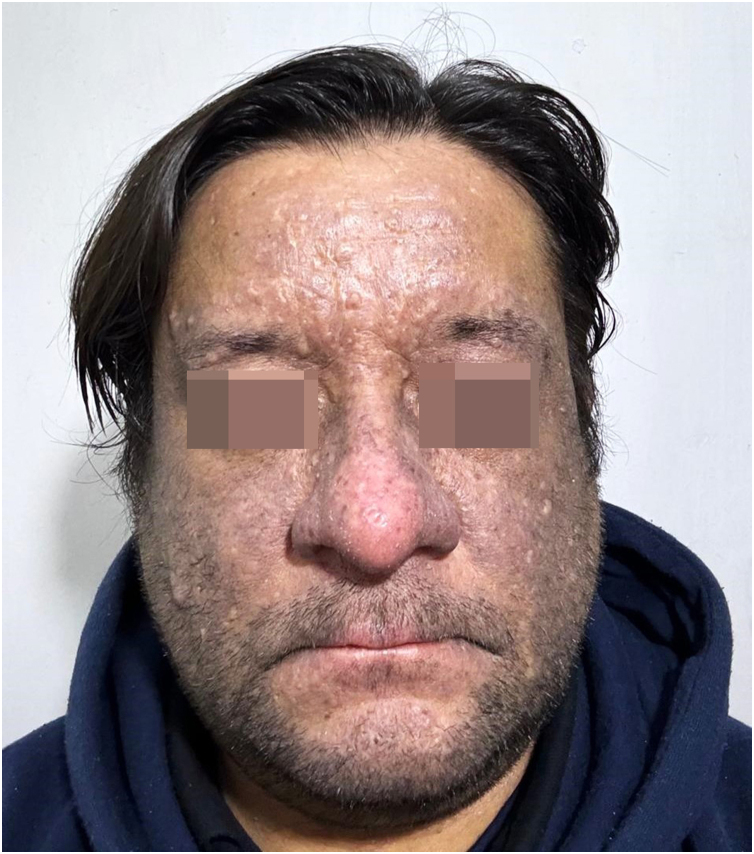
Facial hyperpigmentation after 4 months of adalimumab therapy, showing a slight reduction following the discontinuation of golimumab.

The majority of documented cases involving immune-mediated cutaneous eruptions associated with TNF-alpha inhibitors commonly represent the onset of psoriasis or psoriasiform drug reactions.^1^ However, albeit rare, there is an increasing number of reports associating TNF-alpha inhibitors with the onset of lichenoid eruption.^3^ Among these reported cases, the frequency of occurrences was highest with infliximab, followed by etanercept, adalimumab and certolizumab, in decreasing order.^4^, ^5^ No previously reported cases of a lichenoid eruption attributed to the use of golimumab were found in the English or Spanish literature upon investigation.

The pathophysiology of anti-TNF-alpha-induced lichenoid eruption remains unclear. However, some authors propose that TNF-alpha inhibition in specific genotypes may lead to the upregulation of opposing cytokines, such as interferon-alpha. This upregulation could activate T-cells and dendritic cells, triggering an inflammatory response that may induce lichen planus.^6^

Reporting rare adverse effects of these medications, increasingly used in clinical practice, is crucial to establishing pharmacovigilance registries. These registries help to understand the long-term implications of treatment with TNF-alpha inhibitors, which are increasingly used and currently extend beyond the timeframe of randomized controlled trials.

## Financial support

None declared.

## Authors’ contributions

Daniela Alfaro-Sepúlveda: The study concept and design, data collection, or analysis and interpretation of data, writing of the manuscript or critical review of important intellectual content, effective participation in the research guidance, intellectual participation in the propaedeutic and/or therapeutic conduct of the studied cases, critical review of the literature and final approval of the final version of the manuscript.

Claudio Escanilla: The study concept and design, data collection, or analysis and interpretation of data, writing of the manuscript or critical review of important intellectual content, effective participation in the research guidance, intellectual participation in the propaedeutic and/or therapeutic conduct of the studied cases, critical review of the literature and final approval of the final version of the manuscript.

Fernando Valenzuela: The study concept and design, data collection, or analysis and interpretation of data, writing of the manuscript or critical review of important intellectual content, effective participation in the research guidance, intellectual participation in the propaedeutic and/or therapeutic conduct of the studied cases, critical review of the literature and final approval of the final version of the manuscript.

Dominga Peirano: The study concept and design, data collection, or analysis and interpretation of data, writing of the manuscript or critical review of important intellectual content, effective participation in the research guidance, intellectual participation in the propaedeutic and/or therapeutic conduct of the studied cases, critical review of the literature and final approval of the final version of the manuscript.

## Conflicts of interest

None declared.
